# Lean Six Sigma Approach for Reducing Length of Hospital Stay for Patients with Femur Fracture in a University Hospital

**DOI:** 10.3390/ijerph18062843

**Published:** 2021-03-11

**Authors:** Arianna Scala, Alfonso Maria Ponsiglione, Ilaria Loperto, Antonio Della Vecchia, Anna Borrelli, Giuseppe Russo, Maria Triassi, Giovanni Improta

**Affiliations:** 1 Department of Public Health, University of Naples “Federico II”, 80131 Naples, Italy; ariannascala7@gmail.com (A.S.); ilaria.loperto@gmail.com (I.L.); triassi@unina.it (M.T.); ing.improta@gmail.com (G.I.); 2 Department of Electrical Engineering and Information Technology, University of Naples “Federico II”, 80125 Naples, Italy; 3 Hospital Directorate, “San Giovanni di Dio e Ruggi d’Aragona” University Hospital of Salerno, 84125 Salerno, Italy; a.dellavecchia@sanita.it (A.D.V.); acquarama@libero.it (A.B.); 4 Hospital Directorate, National Hospital A.O.R.N. “Antonio Cardarelli” of Naples, 80131 Naples, Italy; ariete_gr@libero.it; 5 Interdepartmental Center for Research in Healthcare Management and Innovation in Healthcare (CIRMIS), University of Naples “Federico II”, 80131 Naples, Italy

**Keywords:** Lean Six Sigma, public health, healthcare quality, process improvement, length of stay

## Abstract

Surgical intervention within 48 h of hospital admission is the gold standard procedure for the management of elderly patients with femur fractures, since the increase in preoperative waiting time is correlated with the onset of complications and longer overall length of stay (LOS) in the hospital. However, national evidence demonstrates that there is still the need to provide timely intervention for this type of patient, especially in some regions of central southern Italy. Here we discuss the introduction of a diagnostic–therapeutic assistance pathway (DTAP) to reduce the preoperative LOS for patients undergoing femur fracture surgery in a university hospital. A Lean Six Sigma methodology, based on the DMAIC cycle (Define, Measure, Analyze, Improve, Control), is implemented to evaluate the effectiveness of the DTAP. Data were retrospectively collected and analyzed from two groups of patients before and after the implementation of DTAP over a period of 10 years. The statistics of the process measured before the DTAP showed an average preoperative LOS of 5.6 days (standard deviation of 3.2), thus confirming the need for corrective actions to reduce the LOS in compliance with the national guidelines. The influence of demographic and anamnestic variables on the LOS was evaluated, and the impact of the DTAP was measured and discussed, demonstrating the effectiveness of the improvement actions implemented over the years and leading to a significant reduction in the preoperative LOS, which decreased to an average of 3.5 days (standard deviation of 3.60). The obtained reduction of 39% in the average LOS proved to be in good agreement with previously developed DTAPs for femur fracture available in the literature.

## 1. Introduction

Studies regarding femur fracture are often related to senior patients, especially females [1], who are commonly affected by osteoporosis [2] due to their bone fragility and are therefore more likely to suffer the aforementioned fracture. Fractures of the femur in the elderly are a very frequent event and lead to serious consequences because, generally, in patients over 65 years old, the most frequent fracture is that of the head of the femur; we speak in these cases of fracture at the neck of the femur and pertrochanteric fracture. This type of fracture severely limits the mobility of the limb, which is already compromised in these patients, and can have even more serious consequences, leading even to the death within a year of those who have suffered the trauma [3,4]. Early treatment of femoral neck fracture by surgery is fundamental in elderly patients, because it reduces the risk of mortality and disability. Most of the available guidelines, in fact, recommend surgery within the first 24 h, and no later than 48 h [5,6], provided that the increase in the preoperative waiting time is correlated with the increase in the onset of complications and particularly mortality in the medium term. However, national evidence in Italy demonstrates that there is still a need to provide timely intervention for this type of patient, especially in some regions of central southern Italy [7]. Indeed, despite the fact that improvements have been registered in some Italian regions, going from 40.21% of interventions being timely in 2012 to 66.12% in 2018 (with over 18,000 patients benefiting from timely surgery), inter- and intra-regional heterogeneity remains important, with percentages of early interventions ranging from 30% to 75%, i.e., both well below and well above the national recommended threshold of 60%. This is interpreted as a reflection of the coexistence of healthcare structures that are timely in terms of reducing the waiting time for femur fractures alongside structures that are still far below the international standard, which is over 90% for elderly patients [7].

This evidence makes it urgent for healthcare organizations to improve their processes, and move towards increasing the quality of the provided health services. Among several process improvement strategies, ranging from simulation models [8,9,10,11] to quality management principles [12,13,14,15], Lean Six Sigma (LSS) has proved to be a successful approach in the healthcare context by combining the power of statistical analysis, which is typical of the Six Sigma, together with the Lean Thinking principles of “zero waste” [16]. Initially used only in the manufacturing industry, LSS has also grown in popularity as a managerial methodology in the healthcare sector, with several applications over the past 20 years [17,18,19,20]. Improta et al. (2018) applied the LSS methodology in the area of clinical medicine at Federico II University Hospital in Naples in order to reduce the risk of healthcare-associated infections (HAI). The result was a decrease in the percentage of colonized patients from 0.36% to 0.19% [21]. The LSS methodology was successfully applied in several hospitals to reduce medication errors [22,23,24]. Mahesh et al. (2018) used the LSS methodology, employing the Define, Measure, Analyze, Improve and Control (DMAIC) cycle, to reduce patient wait times at the Cardiology Out-Patient Department (OPD) of the Multi-Specialty Hospital in Bangalore [25]. Over the years, many researchers have used the LSS methodology to analyze clinical pathways in different branches of medicine. Sayeed et al. (2018) applied the principles of LSS to implement a hip fracture integrated care pathway developed to reduce the percentage of patients operated beyond 48 h of admission [26]. The DMAIC approach was also used to improve the effectiveness and efficiency of the care process of patients undergoing knee replacement surgery due to osteoarthritis. Thanks to the application of fast-track surgery, the average LOS was reduced by 19.9% [27]. Improta et al. (2017) also applied LSS principles to reduce costs related to prosthetic knee replacement surgery. Specifically, they identified variables influencing LOS prolongation and implemented corrective actions to improve the process of care, resulting an average decrease in LOS of 42% [28]. LSS also proved to be a promising approach for the improvement of the femur fracture care pathway: McNamara et al. (2014) focused their study on the improvement of the care process for patients with femur neck fractures, in particular in the surgical theater, and its genesis and duration [29], while Improta et al. (2019) and Ricciardi et al. (2019) focused on the reduction of preoperative LOS and total LOS after the implementation of a DTAP for femur fracture in an Italian hospital [30,31].

This work is intended to expand and deepen a previous study [32] conducted at the university hospital “San Giovanni di Dio e Ruggi D’Aragona” of Salerno within the project “Femur: zero wait”, which adopted LSS to implement a Diagnostic Therapeutic Assistance Path (DTAP) to reduce the preoperative length of stay (LOS) of over-65-year-old patients undergoing surgery for fracture of the femur neck. The study analyzes the impact of the LSS-guided improvements and of the implemented DTAP on several process variables over a longer time span compared to our previously published conference paper. In this way, we confirm and strengthen what has previously been shown about the implementation of DTAP in terms of reduction of the preoperative LOS. Finally, the study discusses a comparison between the presented results and those obtained by implementing analogous clinical pathways, with particular focus on the pathway developed at the Italian national hospital A.O.R.N. Cardarelli of Naples, as reported in [30].

## 2. Materials and Methods

As mentioned, this study represents an extension of our previous work [32], with deeper data analysis over a longer time span. The study was carried out at the Complex Operative Unit of Orthopedic and Traumatology at the “San Giovanni di Dio e Ruggi d’Aragona” university hospital of Salerno (Italy). The LSS DMAIC cycle was adopted to identify critical issues for quality and possible solutions to improve the care process. Each phase is described in detail below.

### 2.1. Data Collection

Data were retrospectively collected on two different groups of patients who had undergone femur fracture surgery before (6 years’ timespan: 2010–2015) and after (5 years’ timespan: 2016–2020) the implementation of the DTAP. The former group included 468 patients, the latter 365.The hospital information system (QUANI SDO) (BIM Italia Srl, Milan, Italy) of the hospital was used to gather data regarding the following anamnestic, demographic and clinical variables related to each patient:

- Gender (male/female);
- Age (65 ≤ Age ≤ 75; 75 < Age ≤ 90; >90)
- Date and time of admission;
- Date and time of surgery;
- Date and time of discharge;
- Hypertension (Yes; No);
- Diabetes (Yes; No);
- Cardiovascular diseases (Yes; No);
- Respiratory diseases (Yes; No);
- Kidney diseases (Yes; No);
- Anaemia (Yes; No);
- Bleeding during surgery (Yes; No).

### 2.2. DMAIC Cycle

The methodological approach adopted in this study was in accordance with the LSS DMAIC cycle and follows its typical workflow, as widely described in the literature and in previous works of the authors [24,25,28], which is divided into five phases: (i) Define phase, where the objective and the Critical to Quality (CTQ) are identified; (ii) Measure phase, where the current process performance is measured; (iii) Analyze phase, where the collected data from the current process are analyzed to identify the main causes of deviation of the CTQ from optimal process conditions, and corrective actions are proposed; (iv) Improvement phase, where the corrective actions and solutions are implemented; (v) Control phase, where the performance of the improved process is monitored and compared with the starting conditions. Details on each phase are described in the following.

#### 2.2.1. Define

The objective, i.e., the “reduction of the preoperative LOS for patients undergoing femur fracture surgery”, as well as the CTQ, i.e., the preoperative LOS, were identified. All aspects of the project are reported in the following project chart:

1. Project title: LSS approach to reduce preoperative LOS through a Diagnostic-Therapeutic-Assistance Path at “San Giovanni di Dio e Ruggi d’Aragona” University Hospital.
2. Question: inappropriate prolongation of preoperative LOS.
3. CTQ: preoperative LOS, measured in days.
4. Target: Realize corrective measures in order to reduce the CTQ increase the percentage of people undergoing surgery within 48 h of admission.
5. Deliverables: increase in admissions and decrease in LOS, improvements in patients’ outcome.
6. Timeline:
  - Define: January–February 2016.
  - Measure: March–April 2016.
  - Analyze: May–August 2016.
  - Improve: September 2016.
  - Control: October 2016–June 2020.
7. In scope: femur surgery. Complex Operative Unit (C.O.U.) of Orthopedic and Traumatology at the “San Giovanni di Dio e Ruggi d’Aragona” University Hospital of Salerno.
8. Out of scope: all other structures and interventions.
9. Financial: no funding was provided to chase the target.
10. Business need: creation of a pathway making it possible to speed up the surgery process.
11. In addition, a SIPOC (Supplier, Input, Process, Output, Customer) scheme was built to clarify the main process characteristics and the scope of the project.

#### 2.2.2. Measure

The aim of this phase was to measure the performance of the current process i.e., before the implementation of the DTAP. In detail, a retrospective measurement of the preoperative LOS (i.e., the identified CTQ of the project) was carried out on a sample of 468 elderly patients (age > 65) diagnosed with fracture of the neck of the femur over a period ranging from 1 January 2010 to 31 December 2015. The preoperative LOS is measured as the number of days from the date of the patient’s hospital admission to the date of the surgical intervention. The results are visualized on a run chart, where the preoperative LOS is reported for each hospitalized patient (i.e., for each observation). The run chart was plotted using Microsoft Excel (MS Office 2016 suite) (Microsoft Corp, Redmond, WA, USA). Descriptive statistics (mean, median, standard deviation) were also calculated using the IBM SPSS Statistics Version 26.0 software (IBM Corp, Armonk, NY, USA).

#### 2.2.3. Analyze

At this stage, each step of the starting preoperative process was described and discussed within the multidisciplinary team in charge of the analysis, guided by the healthcare director of the hospital and its staff. Then, a root causes analysis of excessive preoperative LOS was carried out using an Ishikawa diagram.

The diagram focused on two main aspects: “People” and “Process”. These aspects were then differentiated into four categories: “Patients” and “Clinical staff” belonged to the “People” aspect, whereas “Hospital” and “Process” belonged to the “Process” aspect. Specifically, “Clinical Staff” and “Patients” categories reflected the aptitude of clinicians and healthcare staff towards the change in the process routine and the health status of the patients; “Hospital” aspects included the need for specific examinations and functional imaging and chemistry tests provided by the hospital, as well as the execution of additional instrumental exams performed in the hospital, as required by consultancy; “Process” aspects take into account current procedures that can affect the length of hospital stay.

The influence of demographic and clinical factors on preoperative LOS was assessed through a univariate statistical analysis. Before the analysis, the normal distribution of the data was checked with a Kolmogorov-Smirnov test. Since the data were not normally distributed, non-parametric statistical tests were used for the analysis. Gender, age, hypertension, diabetes, cardiovascular disease, respiratory disease, kidney disease, anemia and bleeding during surgery were considered to be independent variables, and the preoperative LOS was considered to be the dependent variable. For the comparison between groups, the U Mann–Whitney test was used for dichotomous categories and Kruskall–Wallis test for non-dichotomous ones. The significance level (α) of the statistical tests was 0.05 (confidence level of 95%). The IBM SPSS statistics 20 software was used to perform the statistical data analysis.

#### 2.2.4. Improve

In the improvement phase, all the observations, analyses, and discussions emerging during the previous phases were examined and re-assessed by the project team during brainstorming sessions in order to elaborate corrective actions in order to improve the process under study. In this phase, the national guidelines and international recommendations were taken into consideration as a reference for designing a new DTAP for improved management of elderly patients diagnosed with femur fracture before, during, and after surgery, with the final aim of guaranteeing a timely and adequate intervention.

#### 2.2.5. Control

The control phase, in accordance with the DMAIC methodology, was carried out to ensure the value of the new protocol introduced. In particular, data on a sample of 365 elderly patients diagnosed with fractures of the femur after the implementation of the DTAP were retrospectively collected for the period from 1 January 2016 to 30 June 2020. and displayed on a run chart, where preoperative LOS was graphed against each observation. The run chart was plotted using Microsoft Excel (MS Office 2016 suite). Descriptive statistics (mean, median, standard deviation) were calculated for the whole dataset and for each sub-group as defined in the analysis phase (gender, age, hypertension, diabetes, cardiovascular disease, respiratory disease, kidney disease, anemia and bleeding). Then, a U Mann–Whitney test with a significance level (α) of 0.05 (confidence level of 95%) was performed to compare the preoperative LOS before and after the implementation of the DTAP and establish whether a significant reduction in the LOS has been achieved. The overall reduction in the preoperative LOS was also summarized using a boxplot. IBM SPSS statistics 20 software was used to perform the statistical data analysis.

In this phase, an additional assessment of the implemented DTAP is proposed. In particular, a qualitative comparison with analogous pathways available in the literature is provided. Results in terms of reduction in the LOS are shown, compared and critically discussed, highlighting strengths and limitations of the proposed DTAP with respect to the relevant literature in the field.

## 3. Results and Discussion

The SIPOC scheme built in the Define phase is shown in Table 1. It reports a general overview of the process and its major characteristics.

**Figure 1:**
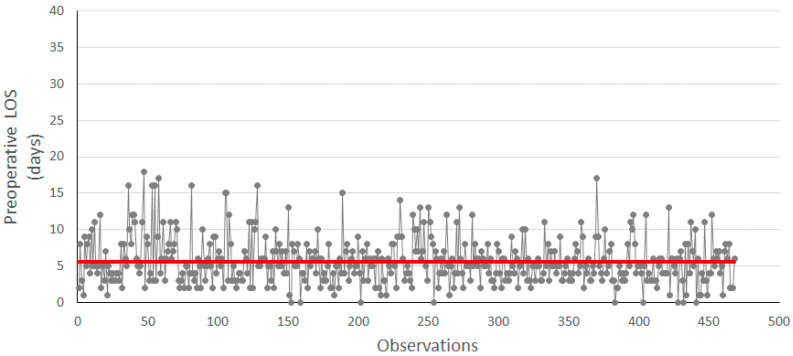
Run chart of preoperative LOS before improvement. The red line indicates the average value of 5.6.

Then, the measurement of the CTQ of the project, i.e., the preoperative LOS, was carried out and reported in the run chart displayed in Figure 1. Preoperative LOS data are displayed in grey, while the red line indicates the average value (in days) of the preoperative LOS.

In the measured period, a patient waited an average of 5.62 days before entering the operating room, with a standard deviation of 3.24. Therefore, in order to comply with the available guidelines recommending surgery within two days, it was necessary to introduce a corrective action, which resulted in the introduction of the DTAP.

During the analysis phase, a more detailed description of the starting process was provided, with a specific focus on the actions and procedures taken during the preoperative management of elderly patients undergoing femur surgery.

The starting preoperative process can be summarized in the following steps:

1. Assignment of the yellow code in the triage phase;
2. Clinical evaluation and physical examination by the emergency room doctor;
3. Execution of instrumental tests as required (X-ray);
4. Hospitalization if femur fracture is confirmed;
5. Performing routine blood chemistry, CBC (cell blood count), D-dimer, chest, hip and lower limb X-ray, ECG;
6. Orthopedic, cardiological, pneumological, geriatric and internist consultancies;
7. Execution of additional instrumental tests as required by consultancy;
8. Drug therapy, such as thrombosis prophylaxis and electrolyte balance
9. Clinical anesthetic evaluation.

**Figure 2:**
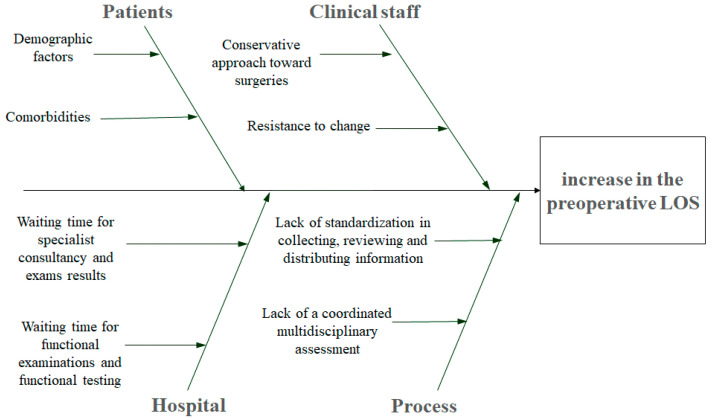
Ishikawa fishbone diagram.

Within these processes, four major causes of prolonged LOS were identified, and these are represented in the Ishikawa diagram (Figure 2), along with related secondary causes.

After the description and discussion of the initial preoperative process, two main organizational reasons for prolonged preoperative LOS were identified: the lack of a coordinated multidisciplinary assessment, and the waiting time for consultancy and examinations. It the organization of regular and periodic multidisciplinary meetings was then suggested, as well as the adoption of a multidisciplinary approach starting from the early hospitalization of the patient. In the preoperative phase, after the hospitalization of the patient, it was recommended that a trade-off be established between the complete and thorough stabilization of the patient and timely surgical intervention, since delays due to the stabilization of the patients could increase the risk of complications after the surgery. In this regard, it was suggested that clear and day-by-day updated objectives be established for the clinical stabilization of patients before surgery. In addition, it was highly recommended that adequate procedures for preventing complications that can occur in successive phases of the process be performed during the emergency phase, with particular regard to: the assessment and control of the pain, especially during the mobilization of the patients undergoing imaging or chemistry examinations; the monitoring of the state of hydration through the infusion of electrolytic or physiologic solutions in the emergency room, since most patients with femur fracture suffer from hydration deficiencies; the early management of pressure lesion already in the emergency room, e.g., avoiding a stay on emergency stretchers of more than 60–90 min; procedures for warming the patient, especially if the stay in the emergency room is longer than 4 h; and the adequate management and consideration of comorbidities, especially in the case of infusion of electrolytic solutions.

As far as the demographic factors and comorbidities are concerned, in order to better understand which of these patient-related variables influenced the preoperative LOS the most, a univariate statistical analysis was carried out (Table 2). Statistically significant *p*-values are highlighted in bold.

Table 2 shows that the variables that most influence LOS are hypertension, cardiovascular disease, and bleeding during surgery (*p*-value < 0.05). The median values of the LOS were also calculated, being equal to 5 days for all categories, with only one exception for the group of patients with kidney disease, which showed a median LOS value of 4.5 days.

**Figure 3:**
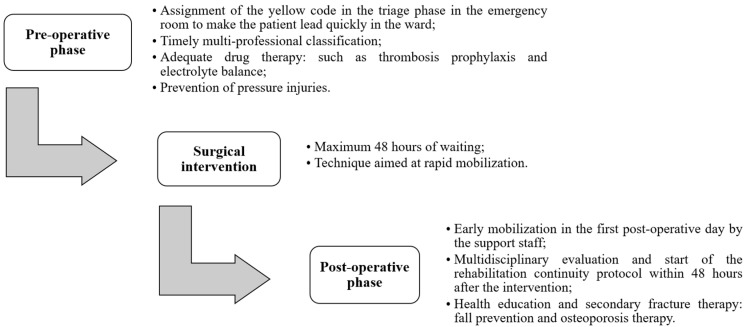
The three main phases of the implemented DTAP.

In the improvement phase, a DTAP was proposed, developed and implemented to ensure a better and faster management of elderly patients diagnosed with fracture of the neck of the femur. The DTAP takes into consideration all the observations and recommendations that emerged in the analysis phase, as well as further strategies for the management of the patient before, during, and after the surgery. The DTAP consists of three stages, as described in our previous publication [32]. Figure 3 shows the three main phases in detail.

It is worth mentioning that the proposed DTAP is analogous to previously developed care pathways at the hospital A.O.R.N. “A. Cardarelli”, as described in [30]. Among them, the presence of a protocol for the rapid transfer of the patient from the emergency department to the orthopedic ward, timely multi-professional (orthopedic, internal medicine, anesthesiology and nursing) assessment, and, not least, early rehabilitation care, are common points for both the pathways implemented at the hospital Cardarelli of Naples and the hospital Ruggi of Salerno, respectively. This observation could represent evidence for the potential translation of the DTAP to other healthcare facilities that share similarities in terms of their organization and structures, as well as facing analogous issues.

**Figure 4:**
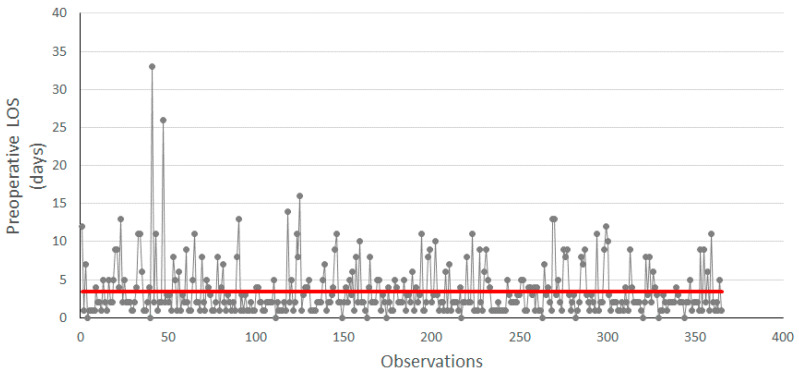
Run chart of preoperative LOS after improvement. Red line indicates the average value of 3.5.

In the control phase, the impact of the introduced DTAP on the preoperative LOS was assessed. The results of the comparison in terms of preoperative LOS before and after the implementation of the DTAP are shown in Figure 4 and Table 3, below.

In particular, Figure 4 shows the measurement of the preoperative LOS after the implementation of DTAP, where the LOS measured for each observation is reported in grey, while the red line indicates the average value in days of the preoperative LOS. The average value of the preoperative LOS is now 3.45, and the standard deviation is 3.60.

When compared to Figure 1, the run chart in Figure 4 shows a decrease in the average LOS. In fact, the average preoperative LOS before DTAP implementation was 5.62 (as in Figure 1), compared to an average of 3.45 after implementation (Figure 4).

The complete statistical analysis demonstrates a significant difference in the preoperative LOS before and after implementation of the new protocol (Table 3). Statistically significant *p*-values are highlighted in bold.

Indeed, Table 3 shows that for all categories, there was a significant percentage decrease in mean preoperative LOS days, with the *p*-value being well below 0.05 for almost all categories. Median values of the LOS post-DTAP were also calculated, being equal to 2 days for all the categories with only one exception for the group of patients with bleeding during surgery, which showed a median LOS value of 8 days. Patients with kidney disease and with bleeding during surgery had the smallest and least significant decreases, of −4.63% and −6.60%, respectively. As far as patients with kidney disease, for whom the reduction of preoperative LOS was not statistically significant, are concerned, it should be considered that these patients are often characterized by comorbidities. Furthermore, patients with kidney disease have a higher incidence of hip fracture and an increased risk of death after fracture [33,34]. For these reasons, better multidisciplinary preoperative characterization is advisable [35]. Therefore, it is important to find a balance between the timing of surgery and the patient’s physical condition. In previous studies, delaying surgery by more than 48 h in elderly patients with hip fractures led to an increase in post-surgery mortality rates. However, a recent study showed that delaying surgery for several days did not negatively impact the incidence of postoperative adverse events [36]. As a consequence of the above, it is believed that this lower reduction in preoperative LOS is not a failure of the methodology applied. As far as bleeding during surgery, on the other hand, is concerned, it can have a multifactorial etiology and it is of more complex characterization, as it could be either the cause or consequence of a longer preoperative LOS (comorbidity, protracted anticoagulant therapy).

**Figure 5:**
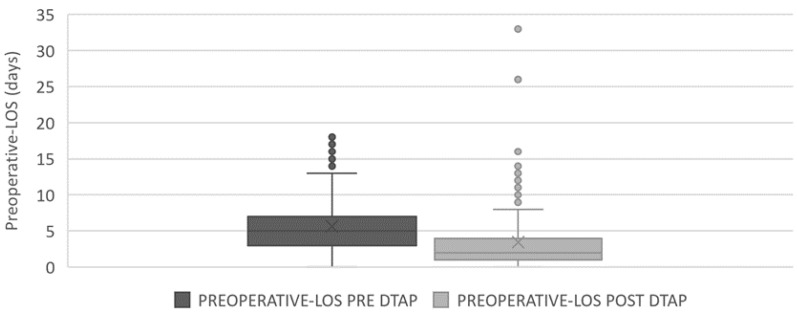
Boxplot of preoperative LOS before and after DTAP.

A boxplot was also used (Figure 5) to show the significant decrease in the overall preoperative LOS.

Furthermore, it is worth observing that the percentage of over-65 patients with femur fracture who underwent femur fracture surgery within 48 h increased from 13.5% to 60.3% after the implementation of the DTAP, reaching the threshold value recommended by the national guidelines (60%). The results are then in accordance with the guidelines of the Italian Ministry of Health regarding the use of quality indicators to measure the performance of national healthcare facilities [37]. Indeed, among the reported indicators, the percentage of over-65 patients with femur fracture who were operated on within 48 h of hospital admission should be above the threshold of 60% in order to ensure appropriate management of elderly patients undergoing femur surgery and reduce the risk of short-, medium- and long-term complications. The obtained results demonstrate that the implemented of the DTAP made a significant contribution in this sense, making it possible to improve the indicator, thus reaching the nationally recommended performance threshold.

Our results also confirm the effectiveness of the proposed clinical pathway in significantly decreasing the LOS for several categories of patients with different clinical conditions as shown (Table 3). Such an impact of the clinical pathways is widely recognized in the literature. Indeed, as outlined in the European Quality of Care Pathways (EQCP)-study on proximal femur fracture [38] performed by the European Pathway Association, an international not-for profit association, they demonstrated not only that care pathways can significantly reduce the LOS in hospital settings, thus having a positive impact on different healthcare outcomes, but also that the LOS, with particular regard to the preoperative LOS, is a crucial indicator that needs to be monitored and taken into great account when designing a quality improvement study [38] and this has been proven not only in the case in the elderly population, but also in the case of the pediatric population [39].

To further assess the effectiveness of the DTAP described here, a brief comparison with three analogous studies implementing a DTPA for femur fracture is reported Table 4.

The proposed pathway shows comparable results with other DTAP for managing patients undergoing femur fracture surgery. In particular, the obtained reduction of 39% in the preoperative LOS is slightly higher than the one obtained Kosy et al. [40] in a district general hospital in South West England but it is lower when compared to the 54% reduction achieved by Improta et al. [30] at the hospital A.O.R.N. “A. Cardarelli”. However, it is worth mentioning that the comparison shown should be considered in broad terms, and does not represent an analytical evaluation or a validation, since it is affected by multiple other factors. Indeed, it should be taken into account that study designs can be substantially different and, even when a care pathway is effectively developed and implemented, many of the observed results could not be attributed directly to the pathway itself [38]. Despite these limitations, the proposed study presents a general framework for the applicability of quality and efficiency improvement projects through the LSS DMAIC cycle and for the use of LSS to evaluate the effectiveness of DTAPs in hospital settings.

## 4. Conclusions

In this paper, the LSS methodology was adopted to reduce preoperative LOS for patients undergoing femur fracture surgery at the “San Giovanni di Dio e Ruggi d’Aragona” university hospital. The DMAIC cycle was applied to achieve this aim. In the define phase, the main characteristics of the project were defined and represented thanks to a project charter and a SIPOC diagram. In the measure phase, the older process was evaluated on the basis of mean and standard deviation and represented using a run chart. In the analysis phase, the causes of prolonged preoperative LOS were investigated, and statistical analyses were carried out in order to identify the variables that most influenced preoperative LOS. Then, the implemented DTAP was described in the improve phase. Finally, the control phase showed that the achieved results were guaranteed over a long-term period. The significant results obtained show that the average preoperative LOS fell from 5.62 to 3.45 days, with a percentage decrease of 39%. Compared to the relevant literature in the field and to analogous clinical pathways, the proposed study showed interesting and relevant results, providing a helpful guide for quality improvement studies in healthcare settings. However, as emerged from the literature, it is important to highlight that the intrinsic characteristics and conditions of the organization in which the clinical pathway is implemented should be taken into high consideration, since they could significantly affect outputs and outcomes of the process.

## Figures and Tables

**Table 1:** SIPOC table.

Supplier	Input	Process	Output	Customer
Complex Operative Unit (C.O.U.) of Orthopedic and Traumatology	Surgical and medical services	Care process	Faster time of intervention;	Patients;
			Improved outcome for patients.	“San Giovanni di Dio e Ruggi d’Aragona” University Hospital of Salerno.

**Table 2:** Variables influencing preoperative LOS (pre-DTAP).

Variable	Category	N	Preoperative LOS [Mean ± dev std]	*p*-Value
All	All	468	5.62 ± 3.24	-
Gender	Man	138	5.70 ± 2.86	0.281
	Women	330	5.59 ± 3.39	
Age	65 ≤ Age ≤ 75	94	6.34 ± 3.93	0.293
	75 < Age ≤ 90	323	5.51 ± 3.00	
	>90	51	5.000 ± 3.14	
Hypertension	No	369	5.82 ± 3.34	**0.021**
	Yes	99	4.89 ± 2.71	
Diabetes	No	399	5.65 ± 3.20	0.302
	Yes	69	5.48 ± 3.48	
Cardiovascular disease	No	304	5.98 ± 3.31	**0.0004**
	Yes	164	4.96 ± 3.00	
Respiratory disease	No	428	5.63 ± 3.28	0.741
	Yes	40	5.600 ± 2.77	
Kidney disease	No	444	5.670 ± 3.26	0.219
	Yes	24	4.750 ± 2.66	
Anemia	No	337	5.740 ± 3.34	0.395
	Yes	131	5.320 ± 2.954	
Bleeding during surgery	No	347	5.100 ± 2.944	**<0.0001**
	Yes	121	7.120 ± 3.583	

**Table 3:** Statistical analysis of preoperative LOS related to each variable and category.

Variable	Category	LOS Pre-DTAP [Mean ± dev std]	LOS Post-DTAP [Mean ± dev std]	Mean Difference [%]	*p*-Value
All	All	5.62 ± 3.24	3.45 ± 3.59	−39%	**<0.0001**
Gender	Man	5.70 ± 2.86	3.87 ± 4.38	−32.1%	**<0.0001**
	Women	5.59 ± 3.39	3.32 ± 3.32	−40.6%	**<0.0001**
Age	65 ≤ Age ≤ 75	6.34 ± 3.93	4.39 ± 5.51	−30.8%	**<0.0001**
	75 < Age ≤ 90	5.51 ± 3.00	3.31 ± 3.04	−39.9%	**<0.0001**
	>90	5.00 ± 3.14	2.89 ± 2.56	−42.2%	**0.0002**
Hypertension	No	5.82 ± 3.34	3.78 ± 3.98	−35.1%	**<0.0001**
	Yes	4.89 ± 2.71	2.94 ± 2.87	−39.9%	**<0.0001**
Diabetes	No	5.65 ± 3.20	3.62 ± 3.84	−35.9%	**<0.0001**
	Yes	5.48 ± 3.48	2.58 ± 1.75	−52.9%	**<0.0001**
Cardiovascular disease	No	5.98 ± 3.31	3.61 ± 3.51	−39.6%	**<0.0001**
	Yes	4.96 ± 3.00	3.12 ± 3.78	−37.1%	**<0.0001**
Respiratory disease	No	5.63 ± 3.28	3.47 ± 3.66	−38.4%	**<0.0001**
	Yes	5.60 ± 2.77	3.24 ± 3.03	−42.1%	**0.0005**
Kidney disease	No	5.67 ± 3.26	3.40 ± 3.58	−40%	**<0.0001**
	Yes	4.75 ± 2.66	4.53 ± 3.91	−4.63%	0.391
Anemia	No	5.74 ± 3.34	3.97 ± 4.15	−30.8%	**<0.0001**
	Yes	5.32 ± 2.95	2.80 ± 2.64	−47.4%	**<0.0001**
Bleeding during surgery	No	5.10 ± 2.94	2.95 ± 3.19	−42.2%	**<0.0001**
	Yes	7.12 ± 3.58	6.65 ± 4.42	−6.60%	0.569

**Table 4:** Comparison of the results achieved by implementing DTAP for femur fracture in analogous literature works.

Mean Preoperative LOS before DTAP [Days]	Mean Preoperative LOS after DTAP [days]	Difference in Preoperative LOS [%]	Settings	Reference
5.62	3.45	−39%	University Hospital “San Giovanni di Dio e Ruggi d’Aragona” of Salerno (Italy)	this study
6.90	3.15	−54%	Hospital A.O.R.N. “A. Cardarelli” of Naples (Italy)	[23]
not reported	not reported	not reported	British Orthopaedic Association (UK)	[24]
2.00	1.00	−50%	Children’s Hospital, San Diego	[28]
1.87	1.22	−35%	Hospital in South West England (UK)	[29]

## Data Availability

The data presented in this study are available from the authors upon reasonable request.
